# Beta-Glucan as Wall Material in Encapsulation of Elderberry (*Sambucus nigra*) Extract

**DOI:** 10.1007/s11130-019-00741-x

**Published:** 2019-06-07

**Authors:** Małgorzata Sobieralska, Marcin Andrzej Kurek

**Affiliations:** Department of Technique and Food Development, Warsaw University of Life Sciences, Nowoursynowska 159c, 02-776 Warsaw, Poland

**Keywords:** *β*-glucan, Elderberry, Microencapsulation, Anthocyanins

## Abstract

The aim of the study was to investigate the potential of using *β*-glucan as wall material to microencapsulate the elderberry extract. Firstly, the extract was obtained by the water-acetone extraction method to extract mainly anthocyanins from ground dried fruits. The extract was mixed with wall materials: maltodextrin-*β*-glucan mixture and the control sample as a widely used combination of maltodextrin and arabic gum (92.5:7.5). In the examined samples the content of *β*-glucan was 0.5, 1, 2 and 3%. Properties of encapsulated extracts of final powders were measured using particle size and morphology, encapsulation efficiency, color measurement, total anthocyanin and ascorbic acid content (TAC and TAAC) methods. Our results indicated that the *β*-glucan wall material samples had higher process quality compared to control samples. Addition of *β*-glucan insignificantly decreases encapsulation efficiency. Among powders with *β*-glucan content, the powder with 1% *β*-glucan content was characterized by the smallest (24 μm) particle size. The sample with 2% *β*-glucan content had the highest water solubility and polydispersity index. Due to the encapsulation efficiency, moisture content, and water solubility index, the optimum condition of microencapsulation process for elderberry extract was for samples with 0.5% *β*-glucan as wall material content. To conclude, due to high molecular weight of *β*-glucan the higher than 0.5% ratio of *β*-glucan is not recommended for spray-drying method. However, small quantity of health-beneficial *β*-glucan could act as potential encapsulation agent in clean label products to replace Arabic gum.

## Introduction

Elderberry (*Sambucus nigra* L.) is a plant, most common in Europe and North Africa. Nevertheless, the plant is currently wide spreading across the world for rich in essentials fruit and flower production. Through their pharmacological properties elderberry is widely used in domestic medicine [1]. The most commonly known health-beneficial effects are antibacterial and antiviral properties, ability to reducing sugar and lipid concentration, antidepressant and antitumor properties [2, 3]. Furthermore, the infusions prepared from elderflowers and elderberry significantly reduce the oxidative stress [4]. Elderberry is known for the great amount of bioactive compounds, especially high biological activity polyphenols, such as ascorbic acid, flavonols, phenolic acids, and anthocyanins; the latter is also a source of the characteristic black-purple color of berries [3, 5]. Bioactive compounds of *Sambucus nigra* L. can be effectively obtained by concentrating the juice using nanofiltration membranes. After nanofiltration, hypoglycemic activity of elderberry juice increases as compared to unfiltered juice [6]. However, it is worth to submit that the unripe fruits and other parts of the plant (leaves, bark, seeds) contain harmful compounds like cyanogenic glycoside. Their consumption may cause nausea, vomiting, and more severe side effects, including numbness and stupor [2, 7]. However, research on short-term heating elderberry has a positive effect on reducing the risk of allergic reactions, caused by the presence of allergens such as lectins ebulin f and SELfd, with an insignificant reduction of health-beneficial compounds [8].

The high content of anthocyanins among other berries that occur in the largest number in the plant are cyanidin-3-sambubioside and cyanidin-3-glucoside, and in smaller amount cyanidin-3-sambubioside-5-glucoside and cyanidin-3,5-diglucoside [5, 9]. *Sambucus nigra* L. contains around 800 mg cyanidin-anthocyanins in 100 g cultivars [3]. Anthocyanins, chemically classified as flavonoids, have been revealed in many researches as a result of their antioxidant activity, prevention of chronic disease progression such as cancer [7], the anti-influenza and the anti-cold effect [10, 11], reducing oxidative damage to the human body [3, 5, 8], and reducing diabetes and obesity [2].

The factors which contribute to relative instability and low extraction percentages of anthocyanins are pH, storage temperature, chemical structure, oxygen, solvents, the presence of enzymes, concentration, light, flavonoids, proteins, and metallic ions [12, 13]. Over the past few years, there has been a growing interest in microencapsulation as a method to reduce the adverse influence of environmental factors on anthocyanins. For this method, substances are packed in coating materials and formed into micro or nanoparticles [12, 14]. The spray-drying method has been used for this study as the most rapidly successful method for anthocyanins microencapsulation [15]. High number of encapsulation materials are possible to use for coating in this method, both individually and in combination, *e.g.*, polysaccharides and proteins [16]. Recent studies show that higher content of coating material have positive effect on quality of microencapsulation of anthocyanins [17]. The wall material selection can affect the efficiency of the microencapsulation since there is a significant difference between their ability to form films, biodegradability, resistance to gastrointestinal tract, viscosity, solids content, hygroscopicity, and cost [18]. Except for great coating ability, the reason for using *β*-glucan (BG) as wall material in this study was also its health beneficial properties. This dietary fiber, occurring mainly in the barley and oat, intake of 3 g *per* day lowers the risk of diabetes and associated cardiovascular risks significantly [19, 20].

The aim of this study was to exchange widely used mix of maltodextrin and Arabic gum (MD + GA) wall material to (MD)-*β*-glucan mixture. The effect of different ratio of *β*-glucan as wall material on properties of microencapsulated elderberry extract was investigated.

## Materials and Methods

### Materials

Lyophilized elderberry (*Sambucus nigra*) were purchased from a commercial supermarket in Warsaw. Β-glucan from barley *β*-glucan preparation was extracted as in the study described by Kurek et al. [21]. Arabic gum and maltodextrin (DE 18–20) were supplied from Agnex, Poland. All general chemicals used in this study were of analytical grade and provided by Avantor Poch, POLAND.

### Methods

#### Elderberry Extract Preparation

Anthocyanin extract was obtained by lyophilizing 100 g of elderberry fruits and then grinding them in a laboratory mill. The obtained granulate was sieved to a maximum size of 0.5 mm. The 30 g of dried material was placed in a dark bottle with 100 ml mix of acetone and water (80:20). The resulting suspension was then placed in an ultrasonic bath (Tovatech E100H, Elma, USA) at ambient temperature (around 23 °C) for 15 min and then sedimented for 1 h. The suspension was then filtered through a 0.45 μm filter and poured into a separating funnel. The same volume of petroleum ether was added to the funnel and shaken for 5 min. After separation of the fluid layers, the bottom layer was collected and evaporated using a rotary evaporator to the unrecognizable smell of acetone. At the end of the process the minimum refractometric index of extract should be 15° Brix.

### Microcapsules Production

Solutions of coating materials for the microencapsulation were prepared by dissolving in distilled maltodextrin and arabic gum in 200 ml in an amount of 92.5 and 7.5 g for the control sample. In the tested samples in *β*-glucan was the amount of 0.5, 1, 2 and 3% as the wall material ingredient with constant content of solids - 30% due to high viscosity of *β*-glucan solution. The mixtures were heated to 60 °C and stirred on a magnetic stirrer until the polysaccharides were hydrated. To prepare the drying mixture, 40 g of the elderberry extract was successively combined with 120 g of each coating material solution. The mixture was then homogenized for 3 min at 8,000 rpm. The homogenates were dried in a Buchi B-290 spray dryer. The drying process was carried out at 140 °C, the flow of the pump 25%, air flow 60 l/h.

### Properties of Microcapsules

Bulk density of powders was measured by weighing of samples and placing into a graduated cylinder. The bulk density was calculated by dividing the mass of powder by the volume occupied in the cylinder and expressed as g/cm^3^. Moisture content was measured by water content drying method using moisture analyzer RAD WAG MA 50. For the determination of hygroscopicity of each sample, they were weighed to about 1 g. The powder was placed into aluminum caps and then located in a container with saturated (75%) sodium chloride (NaCl). The hygroscopicity was calculated according to Caparino et al. [22] as a percentage or 1 g of adsorbed moisture *per* 100 g of dry solids (g/100 g). To determine water solubility index 1 g microcapsules of each sample was weighed and then dissolved in 100 ml of distilled water under magnetic stirring for 5 min. Samples were centrifuged at 3,000×*g* for 15 min. The obtained supernatant, in the amount of 25 mL, was transferred to a 50 mL beaker and then dried at 105 °C for 24 h. Solubility (%) was calculated by weight difference according to the equation:

$$ Solubility={P}_a-{P}_b/0.25\times 100, $$

P_a_ (g): the mass of the beaker plus sample after dried
P_b_ (g): the initial mass of the weighed beaker

### Particle Size and Morphology

The morphology of the microcapsules was visually assessed by using Morphologi® G3SE (Malvern Instruments Ltd., Malvern, UK) equipped with a sample dispersion unit. Particle size distribution was calculated as the relative volume of particles in size bands presented in the size distribution curves (Malvern Software v. 5.40, Malvern Instruments Ltd.). Parameters included the D10, D50 and D90 and the mean particle diameter. Polydispersity index was estimated using (D90-D10)/D50) formula. For observation of the morphology of the microencapsulated anthocyanin, Scanning Electron Microscopy was analyzed by a scanning electron microscope (QUANTA 200).

### Encapsulation Efficiency

To evaluate the encapsulation efficiency (% EE), measurement of the total anthocyanin content (TAC) and surface anthocyanins content (SAC) of the microencapsulated powders were used. For the SAC examination, the procedure was the same as in the case of TAC determination, excluding the homogenization. Encapsulation efficiency was calculated according to the equation:

$$ Efficiency=\frac{TAC- SAC}{TAC}\times 100\% $$

TAC: the total anthocyanins contents
SAC: the total surface contents

### Color Measurement

To evaluate the effect of storage on color features of microencapsulated elderberry extract, color of anthocyanin samples were measured immediately after production and after seven days of storage. Color of each powder was measured using a CR-400 colorimeter (Konica Minolta Inc., Tokyo, Japan), and the results were expressed according to the CIELab color space parameters.

### Total Anthocyanin and Ascorbic Acid Content (TAC and TAAC)

TAC and TAAC in the capsules were evaluated one day after preparing and after a week of storage in 25 °C. To investigate the anthocyanin content the pH-differential method was used. Predominant structural forms of anthocyanins present at different pH levels: the colored forms predominate at pH 1.0 and the colorless form at pH 4.5. The absorbance at the 510 and 700 nm was measured in different pH using two buffers which not affected interfering materials [23]. The results are expressed as mg of cyanide-3-glucoside equivalents *per* 100 mg dry weight of microencapsulated elderberry extract powder (mg c3g/kg dmp). For the examination of ascorbic acid and anthocyanin content, 500 mg capsules or 500 ul extract were flooded with 2.5 ml formic acid buffer at pH 3. Samples were homogenized for 30 s and centrifuged. The content of ascorbic acid in capsules was determined using high-performance liquid chromatography using Shimadzu (Kyoto, Japan) system, consisting of a column oven (model CBM-20A), a UV-visible diode-array detector (model SPD-M10), a degasser (model DGU 20A), and a liquid chromatography pump (model LC-20 AD). The column was C18 15 cm long. The conditions for ascorbic acid determination were 95% of formate buffer (A) and 5% methanol (B) with isocratic flow of 1 ml/min. The assay was carried out in three replications. The results were elaborated with reference to the standard and expressed in mg/100 g of powder. The loss during microencapsulation of TAC or TAAC was calculated as a percentage of anthocyanin and ascorbic acid content in the microencapsulated extract (mg/100 g) was examined on the first and seventh day of storage.

### Statistical Analysis

The data were reported as the mean ± standard deviation. The obtained results were subjected to statistical analysis using the Statistica 13.1 program. A one-way analysis of variance ANOVA was applied.

## Results and Discussion

### Physical Properties

The research shows that difference in bulk density could be influenced by the drying parameters, mainly by the inlet air temperature, particle size, texture, and flow properties of freeze-dried powders [24]. The largest bulk density had a sample with the highest amount of *β*-glucan, and the smallest sample with the lowest amount of *β*-glucan. The high bulk density values (0.93 ± 0.001) of *β*-glucan ratio - 3% is related to the molecular weight of this powder (Table 1). The same conclusion was revealed in other studies [14, 24].

**Table 1:** Results of the bulk density, moisture content, hygroscopicity, water solubility and microencapsulation efficiency of microcapsules (average ± standard deviation)

Sample	Bulk density [g/cm^3^]	Moisture content [%]	Hygroscopicity [g/100 g]	Water solubility	Efficiency [%]
0.5% BG	0.89 ± 0.01^a^	1.15 ± 0.001^a^	0.14 ± 0.005^d^	89.86 ± 0.1^b^	93.91 ± 2.72^d^
1% BG	0.92 ± 0.01^d^	1.25 ± 0.042^c^	0.15 ± 0.002^e^	89.14 ± 0.1^a^	89.95 ± 2.02^c^
2% BG	0.91 ± 0.01^b^	1.39 ± 0.002^d^	0.12 ± 0.001^a^	90.18 ± 0.1^c^	85.59 ± 6.15^b^
3% BG	0.93 ± 0.01^e^	1.18 ± 0.001^b^	0.13 ± 0.001^c^	90.02 ± 0.1^bc^	77.97 ± 2.35^a^
MD + GA	0.92 ± 0.01^c^	1.19 ± 0.001^c^	0.13 ± 0.001^b^	90.10 ± 0.1^c^	80.45 ± 1.39^a^

Different letters within the column mean statistically significant differences at *p* ≤ 0.05

There is a significant influence of spray-drying parameters, especially high temperature, on moisture content [25, 26]. Moisture content of the spray-dried powders were different (ranged from 1.15% ± 0.001 for 0.5% BG to 1.39% ± 0.002 for 2% BG ratio powder). However, the water content in the control sample did not differ statistically from the sample with the highest ratio of *β*-glucan powder. The moisture content was higher for the powders with 1 and 2% of *β*-glucan, than in the 3% ratio of *β*-glucan powder. The research on the use of microencapsulation using milk proteins and MD or mixture of MD + BG as wall material indicates, that adding beta-glucan might reduce the moisture content [27]. Mahdavi et al. [14] showed that shorter chains and more hydrophilic groups result in binding with water from the air after drying in Arabic gum and maltodextrin.

The opposite relation with moisture content and hygroscopicity is well observed in the sample with 2% *β*-glucan content (moisture content: 1.388 ± 0.002; hygroscopicity: 0.124 ± 0.0001). The higher water concentration gradient between the product and the surrounding air increases the capacity for water absorption. The same result was submitted by Mahdavi et al. [14] and Tonon et al. studies [24]. Moreover, elderberry extract powders with the lowest *β*-glucan content showed the highest hygroscopicity. The difference in water adsorption may be the result of the different molecular weight of the particles. The lower the molecular weight, the lower the glass transition temperature, which increases the hygroscopicity [14].

The dissolution of the wall materials is relevant to the release of encapsulated anthocyanins. Elderberry extract powders did not differ significantly in terms of the water solubility index, and the high result of solubility of samples was found to be approximately 90%. Amongst samples, the content of 1% *β*-glucan powder was significantly lower (89.14% ± 0.01). The high result of solubility is a result of the high inlet air temperature and use of spary-drying method [25].

### Particle Size and Morphology of Microcapsules

Variance analysis showed that there was statistically significant influence of the *β*-glucan ratio in the dry powder matter on the size of the obtained particles. There was significant dependence for 0.5% (33.233 μm ±0.037) and 3% (33.513 μm ±0.037) (Table 2) *β*-glucan-wall-material ratio for particle size measurement. The size of both powders were above 33 μm when for the rest of the samples, the size was under 29 μm (1% BG - 24.883 μm ±0.027; 2% BG - 29.917 μm ±0.033; MD + GA - 24.443 μm ±0.027). The studies shows that the difference in particle size may be assigned to molecular structure and physicochemical properties as surface activity and molecular weight. The studies on the potential of combination of milk protein sodium caseinate or whey protein concentrate with beta-glucan or MD as wall materials to encapsulate borage oil by spray drying indicates, that wall material components affect the particle size. Milk proteins sodium caseinate (lower molecule size) with beta-glucan possesses more flexible structure, which successfully covered and stabilized a greater interfacial area, which resulted in smaller particle size, than beta-glucan with whey protein concentrate combination (higher molecule size) [27]. Moreover, Tonon et al. [24], in their investigation on microencapsulation of spray-dried açai juice produced with different carrier agents with dextrose equivalent (DE), also observed that particle sizes are related to the molecule size of each carrier agent. The difference of particle size may also have resulted from changes in the parameters during spray-drying, which affect the size of the particles.

**Table 2:** Results of the particle size measurement of microcapsules (average ± standard deviation)

Sample	Particle size [μm]	D10 [μm]	D50 [μm]	D90 [μm]	PDI
0.5% BG	33.23 ± 0.037^d^	13.64 ± 0.015^d^	31.06 ± 0.034^d^	53.74 ± 0.059^d^	1.29 ± 0.001^d^
1% BG	24.88 ± 0.027^b^	10.20 ± 0.011^a^	23.43 ± 0.026^a^	39.43 ± 0.043^a^	1.25 ± 0.001^b^
2% BG	29.92 ± 0.033^c^	12.29 ± 0.014^c^	27.72 ± 0.055^c^	49.50 ± 0.055^c^	1.34 ± 0.001^e^
3% BG	33.51 ± 0.037^e^	13.98 ± 0.015^e^	31.68 ± 0.035^e^	54.66 ± 0.060^e^	1.28 ± 0.001^c^
MD + GA	24.44 ± 0.027^a^	10.45 ± 0.012^b^	23.49 ± 0.026^b^	39.49 ± 0.043^b^	1.23 ± 0.001^a^

Different letters within the column mean statistically significant differences at *p* ≤ 0.05

Statistically significant differences were observed for the polydispersity index in the *β*-glucan-wall-material and control (MD + GA) sample. The highest PDI was observed in 2% *β*-glucan-wall-material ratio (1.341 ± 0.001) and the lowest in the control sample (1.235 ± 0.001). The presence of sugar in microencapsulated extract powders causes rapid moisture absorption and, as a result, lump formation and crystallization of sugar to form a solid bridge. Low polydispersity index value for (MD + GA) powder stems from its higher sugar content compared to powder with *β*-glucan. Powders with the higher dispersibility were found in samples with less tendency for lumping and caking powders, resulting from lower dextrose equivalents ratio [25]. Diffusion-controlled reactions cannot occur in high viscosity matrices in a glassy form, which can have a positive effect on the stability of the anthocyanin powders [28].

**Fig. 1:**
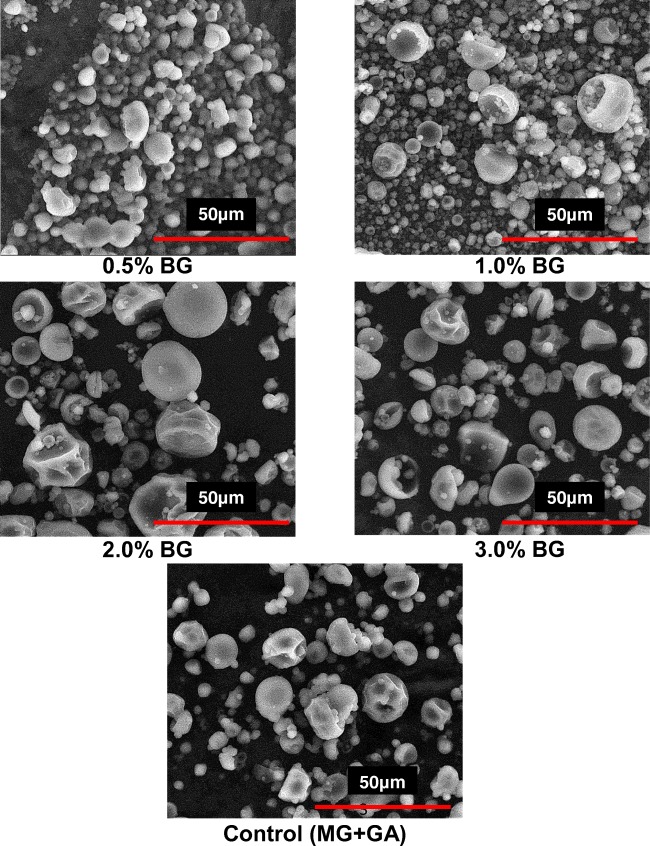
The scanning electron micrographs showing the differences in morphology between the microcapsules

Examination of SEM of encapsulated powders indicated that the powders produced showed morphological properties, typical for materials produced by spray drying as spherical shape and various sizes ranging from 24 to 33 μm (Fig. 1). The 0.5% *β*-glucan ratio microcapsules had the smooth surface and an aggregated spherical, and 3% *β*-glucan ratio microcapsules had higher surface roughness and porosity. The diversity of morphological properties may result from molecular weight of microcapsules with different *β*-glucan ratio. The higher the wall thickness of the microcapsules, the slower they dried, which has a beneficial effect on the creation of a uniform and smooth surface. Moreover, particles with rough surfaces are more sensitive to the oxidation reaction of larger surface areas [14].

### Encapsulation Efficiency

The 3% *β*-glucan encapsulate sample and the control sample did not differ in the statistics and had the lowest value of encapsulation efficiency (3% BG – 77.97% ± 2.350; control sample – 80.45% ±1.3855) (Table 1). However, wall material, as well as the core/wall ratio, affects the encapsulation efficiency. Similar low value of EE for a mixture of MD + GA as wall material was observed in Wilkowska et al. [29] studies where the chokeberry dried juice and wine were encapsulated. Still, the rest of the *β*-glucan samples differed statistically, and the EE grew inversely in proportion to the content of *β*-glucan. These results are different from Mahdavi et al. [14] studies where mixtures of carbohydrates with proteins and polysaccharides (MD + GA) at optimal 25% ratio lead to the higher efficiency as a result of high film-forming capacity and emulsification properties of this mixture. According to these authors, the encapsulated anthocyanins from barberry (*Berberis vulgaris*) in three versions of wall materials, consisting of a combination of maltodextrin and Arabic gum, maltodextrin and gelatin, and maltodextrin obtain encapsulation efficiency between 89.06 to 96.21% where the highest was for MD + GA powder. Nevertheless, the highest EE for elderberry extract powders was for the lowest *β*-glucan ratio powder and amounted to around (93.9% ± 2.717). Similar findings were found in Drozinska et al. [30] studies.

The Li and Shi [27] studies have shown results, that adding high-molecular-weight (range from 31,000 to 237,000 g mol–1) beta-glucan did not improve the EE, comparing to MD with the lower molecular weight (range from 1,250 to 9,000 g mol–1). However, the result shows that the ratio 0.5% of *β*-glucan is the most optimal among other obtained powders including MD + GA. This result corresponded with reports from Robert et al. [31] that the most important variables for the polyphenols encapsulation are the type of encapsulating agent and core to coating ratio.

### Color of Microcapsules

The color measurement indicated a lighter color for the control sample and the sample with the highest *β*-glucan-wall-material ratio. Interestingly, for the parameter a*, the highest value showed a 2% BG ratio sample and high level of parameter b* for samples with 0.5–2% BG ratio (Table 3). Studies show that the high color parameter a* value is justified by a large amount of anthocyanins in extracts [28]. Meanwhile, the lowest values (a* - 9.967 ± 0.015; b* - 2.260 ± 0.01) was observed in control, and 3% BG ratio samples may be attributed to low anthocyanin content. Furthermore, Shishir et al. [25] study point out the influence of maltodextrin level and inlet temperature during spray-drying on powder color. Higher inlet temperature leads to thermal degradation and rapid oxidation, resulting in the lower color of the powders. The difference in color parameters for the powders with *β*-glucan can be the result of different drying parameters, encapsulation efficiency, and total anthocyanin content.

**Table 3:** Results of the colour measurement of microcapsules (average ± standard deviation)

Sample	L*	a*	b*
0.5% BG	77.38 ± 0.21^b^	10.07 ± 0.07^d^	2.48 ± 0.01^c^
1% BG	76.49 ± 0.22^a^	9.84 ± 0.05^b^	2.56 ± 0.02^d^
2% BG	77.88 ± 0.04^c^	11.25 ± 0.02^e^	2.62 ± 0.02^e^
3% BG	79.49 ± 0.01^c^	9.97 ± 0.02^c^	2.26 ± 0.01^a^
MD + GA	79.65 ± 0.02^c^	9.63 ± 0.01^a^	2.35 ± 0.02^b^

Different letters within the column mean statistically significant differences at *p* ≤ 0.05

### Total Anthocyanin Content

The concentration of anthocyanins in both measurements (one day after preparation and a week later) was inversely proportional to the coating material containing *β*-glucan. The first measured anthocyanin powders content were divided into three statistically significant groups: the highest value for 0.5% and 1% BG ratio, mean value for 2 and 3% *β*-glucan ratio, and the lowest for MD + GA (Table 4). Statistically significant differences were observed for the anthocyanin content in samples after a week of storage. The studies show the influence of different amounts of maltodextrin in wall material on the anthocyanins reduction during storage [32]. Beneficial effects of microencapsulation during storage for 10 weeks compared to non-encapsulated anthocyanins was demonstrated in Jafari et al. [28] investigation. Non-encapsulated samples showed a 33% reduction of anthocyanins during 10 weeks of storage and effective protecting properties for microencapsulation of powders. However, for this study, the reduction of anthocyanins was the highest for 3% *β*-glucan ratio and amounted to 20%. The same high result was for the control sample - 18% after one week of storage. Nevertheless, the reduction of anthocyanins was lower for rest of samples. The lower the *β*-glucan ratio, the higher the anthocyanin content after storage.

**Table 4:** Results of the ascorbic acid content and total content of anthocyanins measurement in microcapsules in 1 and 7 day of storage (average ± standard deviation) and percent of degradation during storage

	Total content of anthocyanins [mg/100 g]			Total content of ascorbic acid [mg/100 g]		
Sample	Day 1	Day 7	% of degradation	Day 1	Day 7	% of degradation
0.5% BG	9868.33 ± 106.94^bB^	9408.25 ± 51.60^eA^	5%	21.37 ± 1.95^dB^	17.55 ± 1.27^cA^	12%
1% BG	9753.48 ± 12.81^bB^	8906.92 ± 58.33^dA^	9%	16.61 ± 1.26^cB^	14.08 ± 0.44^bA^	9%
2% BG	9350.85 ± 4.68^aB^	8125.16 ± 111.29^cA^	13%	12.98 ± 0.79^bB^	5.91 ± 0.81^aA^	7%
3% BG	9327.42 ± 315.28^aB^	7371.53 ± 86.22^bA^	21%	9.93 ± 0.15^aB^	5.58 ± 0.29^aA^	6%
MD + GA	8535.16 ± 179.89^cB^	6967.65 ± 22.87^aA^	18%	9.40 ± 0.26^aB^	4.63 ± 0.14^aA^	5%

Different letters within the column mean statistically significant differences at *p* ≤ 0.05

### Total Ascorbic Acid Content

The loss of anthocyanin during spray drying compared with non-microencapsulated extract was significant and ranged from 88 to 95%; the raw extract AA content was 179 ± 3.257 (mg/100 g). Moreover, the loss of ascorbic acid during storage increased with the *β*-glucan content. The lowest residual value was for 2% BG sample and was around 30 times lower than the elderberry non-microencapsulated extract. However, the loss of ascorbic acid content was lower in 0.5 and 1% *β*-glucan ratio powders than in MD + GA powder. Differences in the loss of ascorbic acid depend on the coating material used and the temperature of spray drying. According to other research, Arabic gum and maltodextrin coatings showed the ascorbic acid significantly decreased up to 57% at 120 °C [33]. Similar significant loss was also observed for grapefruit microparticles coated in Arabic gum and bamboo fiber. Phenolic and ascorbic acid contents were also assessed. In this study, spray drying process caused a significant decrease in the content of phenolic compounds in general 42% decrease [34].

## Conclusions

This study successfully demonstrated the superior benefits of using small quantity of *β*-glucan for improving the encapsulation efficiency and stability of elderberry anthocyanin extracts, compared to the usage of maltodextrin with Arabic gum microcapsules. The lowest values of the moisture content were for the 0.5 and 3% *β*-glucan ratio powders. The addition of *β*-glucan positively contribute to improving polydispersity index. However, the highest *β*-glucan ratio was characterized by the lowest efficiency and worse morphology of the microcapsules, which has resulted in higher degradation of anthocyanins and ascorbic acid content after one week of storage. This results from high molecular weight of 3% *β*-glucan ratio powder, which is not recommended for spray drying method. Nonetheless the 0.5% β-glucan ratio is recommended for more efficient microencapsulation due to the encapsulation efficiency, storage loss, ascorbic acid, and anthocyanin total content characteristics. However, while the higher content of maltodextrin or Arabic gum is undesirable, the higher *β*-glucan content as its replacement is favorable due to its health-beneficial effect on human body.

## Abbreviations

BG: Beta-glucan
EE: Encapsulation efficiency
MD: Maltodextrin
MD + GA: Mix of maltodextrin and Arabic gum
DE: Dextrose equivalent
AA: Ascorbic acid
